# Genetic and Phylogenetic Characterization of the M Gene of Influenza A Virus Isolated from Iranian Patients

**Published:** 2019-03

**Authors:** Farida BEHZADIAN, Elham MOASSER, Parviz OWLIA, Horieh SADERI

**Affiliations:** 1. Research Center for Biosciences and Biotechnology, Malek-Ashtar University, Tehran, Iran; 2. Molecular Microbiology Research Center, Shahed University, Tehran, Iran

**Keywords:** Influenza A virus, Iran, M gene, Phylogenetic analysis, Real-time reverse transcription PCR (RT-PCR)

## Abstract

**Background:**

A few studies have been done on the molecular analysis of Iranian influenza A isolates M gene.

**Methods:**

In 2014, nasal swabs collected from outpatients with clinical symptoms in the hospital clinics of Tehran, Iran were subjected for influenza detection and subtyping using Real-Time RT-PCR. Sequence and phylogenetic analysis performed on four randomly selected isolates from each subtype (H1N1 and H3N2) using neighbor-joining method.

**Results:**

Phylogenetic dendrograms drawn based on M nucleotide sequence of H1N1 isolates showed close relatedness with Omanian isolates while the most isolates of H3N2 have clustered with Kuwait isolates and isolates from outside of geographical location. Amino acid sequence analysis showed S31N substitution in all isolates rendering the virus resistant to adamantanes.

**Conclusion:**

This study determined the sequence identity and phylogenetic relatedness of M gene sequence got from Iranian influenza A isolates to elucidate the modality of relationship of this gene in comparison with its counterparts from other regions.

## Introduction

Currently, two Influenza A virus subtypes, H1N1 and H3N2, are circulating among humans. Influenza A viruses are known as a major cause of acute respiratory disease worldwide. Around 1 billion cases of seasonal influenza infection occur each year, with around 3–5 million cases of severe illness, and 300000–500000 deaths (1, 2). Antiviral drugs are used to reduce the duration of disease, viral shedding, rate of hospitalization and death in Influenza A virus-infected persons (3). These drugs are also used for prevention of influenza virus infection (4).

The seventh RNA segment of influenza A virus code two M proteins. While M1 is matrix protein and plays an essential role in virion assembly, budding and release, M2 is ion channel in the envelope and plays important role in virus replication cycle. M2 is activated by low pH of the endosome and conduct protons across the envelope, which results in the acidification of the viral interior. This acidification weakens electrostatic interaction between M1 and ribonucleoprotein (RNP) complexes and releases RNP into cytosol. M2 of influenza A virus is a 97-residue protein and composed of extracellular N-terminal (residue 1–23), transmembrane (residue 24–46), and intracellular C-terminal (residue 47–97) segments (5). Some of amino acid substitutions in the transmembrane segment (position 26, 27, 30, 31, 34, 38 or 41) were shown in amantadine-resistant strains of influenza A virus (4). Adamantanes, amantadine and rimantadine, are M2 protein inhibitors known for many decades to inhibit most influenza A viruses (3, 6). Unfortunately, resistance to amantadine and to its 10-fold more active derivative, rimantadine, develops with increased use in humans and animals. Nowadays, several new kinds of antiviral compounds are being designed and developed against influenza viruses, which block the other critical steps of the viral life cycle (2, 6).

Several articles have been published regarding phylogenetic and molecular analysis of the influenza A virus M gene, isolated from different countries. However, there are a few studies from Iran that focused on M2 sequences (7, 8). Here, we performed a study on the molecular and phylogenetic characterization of the whole M fragment including M2 gene of human influenza A viruses (H1N1 and H3N2) isolated from Iranian patients.

## Materials and Methods

### Isolation of Influenza viruses

Influenza viruses were isolated by standard methods from nasal swabs of outpatients with clinical symptoms in the hospital clinics of Tehran, Iran, during fall and winter 2014. Briefly, the nasal swab samples were tested for presence of Influenza A, H1N1 and H3N2 subtypes using Real-Time Reverse Transcription PCR (RT-PCR) according to instructions recommended by WHO (9). Virus from positive samples was propagated and isolated in MDCK cells. Hemaggluti-nation assay (HA) was performed using a 0.5% suspension of the chicken erythrocytes to confirm virus growth. Positive cultures were harvested and stored at −70°C until further analysis. Four H1N1 and four H3N2 positive samples were randomly selected and subjected for next stages.

### Amplification of M gene

Viral RNA was extracted from 300 μl of the supernatant of cell cultures for each sample using a commercial viral nucleic acid extraction kit (YTA, Iran) and eluted in 20 μl DEPC treated water. Specific primers for amplification of complete M fragment of both genotypes of human influenza viruses, H1N1 and H3N2, were designed using the BioEdit software (version 7.2.5). The list of primers and their characteristics are shown in Table 1. The one-step RT-PCR was done using YTA master mix according to the manufacturer’s recommendation, under the following profile: reverse transcription at 50 °C for 30 min, reverse transcriptase termination at 95 °C for 15 min and HotStart Taq amplification in 40 cycles (95 °C for 20 sec, 58 °C for 40 sec, and 72 °C for 1 min) concluded by a final extension step at 72 °C for 10 min. PCR products were analyzed by gel electrophoresis on a 1.5% agarose gel and then purified by a GF-1 PCR Clean-up Kit (Vivantis, Malaysia) according to the manufacturer’s instruction prior to sequencing.

**Table 1:** Sequences of primers used for amplification and sequencing analysis of M gene of human influenza A viruses

***Influenza Subtype***	***Primer sequence (5′→3′)***		***Tm (°C)***	***PCR product (bp)***
H1N1	Forward	GATGAGTCTTCTAACCGAGG	45	982
	Reverse	TTTACTCTAGCTCTATGTTGACA		
H3N2	Forward	TATTGAAAGATGAGCCTTCTAA	45	992
	Reverse	TTTACTCCAACTCTATGCTGACA		

### Sequencing

The primers used for sequencing were the same used for amplification of M fragment. Sequencing was done in both directions using a BigDye® Terminator v3.1 Cycle Sequencing Kit and ABI PRISM® 3700 DNA Analyzer Sequencer (Applied Biosystems, USA) at sequence laboratories of First BASE Laboratories, Malaysia. All sequences have been deposited in the GenBank database under accession numbers; from KT206209 to KT206212 for H1N1 isolates and from KT220422 to KT220425 for H3N2 isolates.

### Phylogenetic analysis

Phylogenetic trees of M genes were constructed using the neighbor-joining method within MEGA 5.0 using Kimura two-parameter method. The stability of nodes and tree branching was determined by bootstrapping using 1000 repeats of drawing.

## Results

The 982 and 992 bp fragments amplified from the M gene of H1N1 and H3N2 subtypes, respectively. No addition or deletion of nucleotides was found in the sequence of all eight studied viruses, although, the multiple point mutations were found through the M gene. The M nucleotide sequence of H1N1 viruses revealed a similarity of 98.47%–99.08% with reference vaccine strain. This percentage of similarity ranged from 98.77% to 99.28% in the M nucleotide sequence of H3N2 viruses in comparison with reference vaccine strain.

At amino acid level the deduced sequence of M1 and M2 proteins from H1N1 isolates revealed 98.81% and 96.9%–98.96% identity, respectively. This similarity for M1 and M2 proteins of H3N2 isolates was 99.90% and 98.96%–99.90% respectively.

**Fig. 1:**
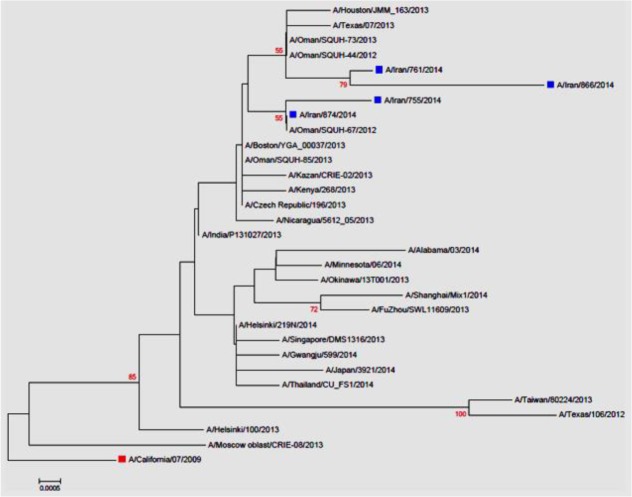
Phylogenetic tree for four Iranian H1N1 matrix gene sequences. Iranian viruses and vaccine strain are shown with blue and red rectangular, respectively

Molecular analysis of the matrix genes of the isolated viruses was therefore done to compare the Iranian isolates with contemporary H1N1 and H3N2 viruses isolated in other countries. The phylogenetic relationships of the M genes were compared to understand more clearly the evolutionary relationships between the viruses (Fig. 1). In addition, the amino acid sequences of the matrix proteins were compared, particularly in terms of differences in M2 (Table 2) which distinguish resistant variants of H1N1 and H3N2 viruses circulating during that period.

**Table 2:** Amino acid changes in the M2 protein related to adamantane resistance

***Virus isolates***		***M2 amino acid profile***							
Subtype	Strain	26	27	30	31	34	38	41	44
H1N1	A/Tehran/755/2014	L	V	A	N	G	L	W	D
H1N1	A/Tehran/761/2014	L	V	A	N	G	L	W	D
H1N1	A/Tehran/866/2014	L	V	A	N	G	L	W	D
H1N1	A/Tehran/874/2014	L	V	A	N	G	L	W	D
H3N2	A/Tehran/217/2014	L	V	A	N	G	L	W	D
H3N2	A/Tehran/221/2014	L	V	A	N	G	L	W	D
H3N2	A/Tehran/244/2014	L	V	A	N	G	L	W	D
H3N2	A/Tehran/247/2014	L	V	A	N	G	L	W	D

Phylogenetic tree constructed based on the M nucleotide sequences of H1N1 isolates shows that Iranian isolates are clustered with the viruses from other Middle East country, Oman, which isolated during previous 2 influenza seasons, i.e. 2012–2013 and 2013–2014 (Fig. 1). They were also clustered with viruses isolated in other parts of the world during 2014–2015 seasons.

**Fig. 2:**
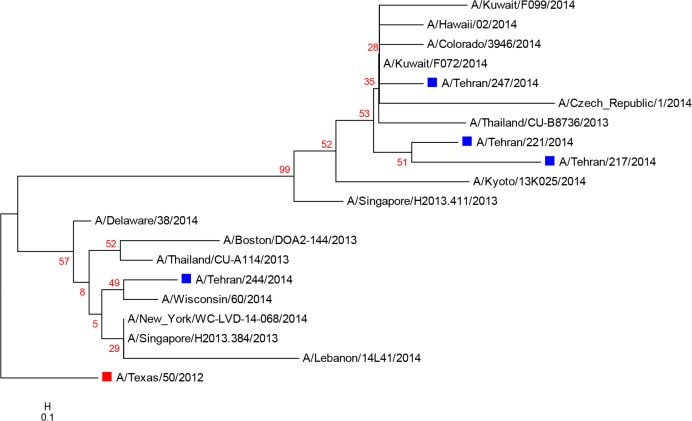
Phylogenetic tree for four Iranian H3N2 matrix gene sequences. Iranian viruses and vaccine strain are shown with blue and red rectangular, respectively

Phylogenetic tree analysis of M gene nucleotide sequences of H3N2 viruses reveals two defined clades (Fig. 2). In the upper clade, three of the four 2014 Iranian isolates clustered with the viruses isolated during the 2014–2015 influenza season in Kuwait. The rest one Iranian isolate clustered with a sequence from Lebanon in second clade. The M genes of Iranian isolates were more closely related to those of the 2014 isolates. Point mutations in M2 sequences of the matrix genes in human influenza A virus isolates were compared between patients and vaccine isolates. All Iranian H1N1 and H3N2 studied isolates possessed the most frequently adamantane-drug resistance mutation resulted in the amino acid substitution S31N in the M2 protein.

## Discussion

The matrix gene encodes for matrix (M1) and ion channel (M2) proteins both with multiple crucial functions. Since M1 protein plays an essential role in virus assembly and budding, any change in M1 can have deleterious effect on virus survival so its gene is conserved with low evolutionary rate into host-specific lineages (10, 11). The complete sequence of M fragment of all isolates included in this study showed high homology with other reference sequences. However, phylogenetic dendrogram drawn based on these sequences demonstrated that the all of four H1N1 Iranian isolates have close relatedness with the isolate belong to the 2013 epidemic season of Oman, suggesting that the current predominant circulating strain of influenza A (H1N1) viruses in Iran might have possibly derived from viruses circulating in this southern neighbor. Interestingly, the same analysis on H3N2 isolates showed that the most prevalent isolates have clustered well in separate group, outside of geographical location. Whereas M1 protein is conserved, M2 protein is more susceptible to mutations. Single point mutations in the amino acids at positions 26, 27, 30, 31, 34, 38, 41 or 44 of the M2 protein, in helix-helix packing interface of the protein; confer cross-resistance to the adamantine, both amantadine and rimantadine (12, 13). The single point mutation at position 31 (S31N) is common and has been identified as the most frequent adamantine-resistant A (H3N2 and H1N1) viruses distributed across the world (14). Resistance to the adamantane generally occurs more readily than resistance to the other replication inhibitors and the resistant strains have not necessarily occurred during antiviral therapy, but have spread widely even in the absence of such drug pressure. Resistant viruses are genetically stable, virulent, and transmissible (15).

Adamantane-resistant H3N2 influenza A viruses have circulated globally since 2003 (16). However, the first significant increase in the incidence of adamantane-resistant H1N1 influenza A viruses has been reported during the 2006 to 2007 season. The rates of resistance vary with the strain of virus but have generally increased over time (17).

There is no comprehensive molecular epidemiological data revealing the origin and emergence of adamantane-resistant human influenza viruses in Iran and can explain the trend of dissemination of such viruses among Iranian patients. However, some limited studies carried on Iranian isolates got from 2005 to 2008 have demonstrated the prevalence of amantadine-resistance A (H3N2) mutants, all resulted in the amino acid substitution S31N in the M2 protein (7). The present study showed that all strains of H1N1 and H3N2 subtypes contained the amino acid substitutions S31N, as expected. However, other mutations in the transmembrane region of M2 linked with adamantane resistance have not been seen in all studied H1N1 and H3N2 viruses (7, 17, 18).

Adamantanes-resistance markers are combined with sequence data of HA and M genes so it may be required to elucidate the genetic association among HA and M2 genes and/or other genes in the generation and spread of community-circulating such resistant strains.

Due to the dramatic increase in resistant isolates, adamantanes not be used for the treatment of influenza, except in selected circumstances (19). However, providing the M fragment sequence data of viruses isolated from different geographic regions and defining their mutation profile would be very helpful to realize the emerging, spread and evolutionary scenario of human influenza A viruses.

## Ethical considerations

Ethical issues (Including plagiarism, informed consent, misconduct, data fabrication and/or falsification, double publication and/or submission, redundancy, etc.) have been completely observed by the authors.

## Conclusion

This study determined the sequence identity and phylogenetic relatedness of M gene got from influenza A circulating trough flu outbreak 2014 in Iran to elucidate the modality of relationship of this gene in comparison with its counterparts from other geographic regions.
